# Decreasing NF-κB Expression Enhances Odontoblastic Differentiation and Collagen Expression in Dental Pulp Stem Cells Exposed to Inflammatory Cytokines

**DOI:** 10.1371/journal.pone.0113334

**Published:** 2015-01-28

**Authors:** Neda S. T. Hozhabri, M. Douglas Benson, Michael D. Vu, Rinkesh H. Patel, Rebecca M. Martinez, Fatemeh N. Nakhaie, Harry K. W. Kim, Venu G. Varanasi

**Affiliations:** 1 Department of Biomedical Sciences and Center for Craniofacial Research and Diagnosis, Texas A&M University Baylor College of Dentistry, Dallas, Texas 75246, United States of America; 2 Center for Excellence in Hip Disorders, Texas Scottish Rite Hospital for Children, Dallas, Texas 75219, United States of America; Georgetown University, UNITED STATES

## Abstract

Inflammatory response in the dental pulp can alter the collagen matrix formation by dental pulp stem cells and lead to a delay or poor healing of the pulp. This inflammatory response is mediated by cytokines, including interleukin-1β and tumor necrosis factor-α. In this study, it is hypothesized that suppressing the actions of these inflammatory cytokines by knocking down the activity of transcription factor Nuclear Factor–κB will lead to dental pulp stem cell differentiation into odontoblasts and the production of collagen. Here, the role of Nuclear Factor–κB signaling and its reduction was examined during odontogenic behavior in the presence of these cytokines. The results showed a significant increase in Nuclear Factor–κB gene expression and p65 protein expression by interleukin-1β and tumor necrosis factor-α. Nuclear Factor–κB activation in the presence of these cytokines decreased significantly in a dose-dependent manner by a Nuclear Factor–κB inhibitor (MG132) and p65 siRNA. Down-regulation of Nuclear Factor–κB activity also enhanced the gene expression of the odontoblastic markers (dentin sialophosphoprotein, Nestin, and alkaline phosphatase) and displayed an odontoblastic cell morphology indicating the promotion of odontogenic differentiation of dental pulp stem cells. Finally, dental pulp stem cells exposed to reduced Nuclear Factor–κB activity resulted in a significant increase in collagen (I)-α1 expression in the presence of these cytokines. In conclusion, a decrease in Nuclear Factor-κB in dental pulp stem cells in the presence of inflammatory cytokines enhanced odontoblastic differentiation and collagen matrix formation.

## Introduction

Dental pulp stem cells (DPSCs) are tissue-specific mesenchymal stem cells found in the human dental pulp [1]. DPSCs play a vital physiological role in regenerating pulp and dentin by proliferating and differentiating into odontoblasts (ODs), the cells responsible for dentin synthesis. Differentiating DPSCs express specific genes needed for the formation of the dental pulp and dentin, including type I collagen, which forms the basis of the dentin extracellular matrix (ECM). These cells also express alkaline phosphatase, which is required to mineralize the ECM, and dentin sialophosphoprotein (DSPP), which plays a crucial role in dentinogenesis [2, 3, 4].

Carious lesions or dental injuries trigger an inflammatory response in the dental pulp that interrupts DPSC differentiation and pulp homeostasis. This response is characterized by an increase in pro-inflammatory cytokines, including tumor necrosis factor—α (TNFα) and interleukin—1β (IL-1β) [5, 6, 7]. Short-term exposure of both cytokines to DPSCs (<3 days) was shown to increase the expression of DSPP and its proteolytic products, dentin sialoprotein (DSP) and dentin phosphoprotein (DPP) [8, 9, 10, 11]. Yet, prolonged pulp inflammation (>3 days) was shown to interfere with odontogenesis [12, 13]. Studies on collagen production demonstrated decreased collagen synthesis and accumulation in the presence of TNFα and IL-1β; TNFα was shown to promote the degradation of collagen produced by pulp fibroblasts [14]. Further, Yang et al. noted that prolonged exposure of rat DPSCs to IL-1β did not stimulate their odontogenic differentiation and attributed this failure to a lack of DSPP stimulation [11]. Together, these data suggest that prolonged inflammatory signaling in DPSCs impedes odontogenesis and pulp tissue regeneration.

Targeted disruption of intracellular signaling mechanisms mediating the inflammatory response may therefore allow therapeutic interventions that promote pulp tissue regeneration. Both TNFα and IL-1β dose-dependently trigger the activation of nuclear factor-κB (NF-κB) by degrading IκBα via the 26S proteasome [15, 16], thus enabling translocation of p65/p50 into the nucleus, where it can activate the NF-κB transcription [17, 18]. The data suggest that interruption of the NF-κB expression may reduce the effects of these cytokines on inflammation. The 26S proteasome inhibitor MG132 inhibits NF-κB activation by preventing IκBα degradation [19, 20]. This inhibitor has shown anti-inflammatory properties such as reducing pro-inflammatory cytokine production, protecting cells from stress via heat shock proteins, and normalizing neuropeptides associated with inflammation and pain in many inflammatory-related disorders, for example, rheumatoid arthritis and osteoarthritis [19, 20, 21]. Similarly, reducing NF-κB expression [using p65 small interfering RNAs (siRNA) to silence the p65 gene in vitro] led to significant amelioration of inflammatory signaling in cells associated with several diseases including rheumatoid arthritis [22], osteoarthritis [23], esophageal cancer [24], and myelodysplastic syndrome [25]. Yet, such targeted NF-κB disruption has not been studied in human dental pulp stem cells exposed to these inflammatory cytokines; this phenomena will be studied here.

Thus, we hypothesize that a decrease in NF-κB expression in the presence of IL-1β and TNFα will enhance DPSC odontogenic differentiation. The purposes of this study are (1) to determine the dose-dependent effect of TNFα and IL-1β on the DPSC NF-κB expression, odontogenic marker gene expression, and collagen synthesis, and (2) to determine the effect of reduced NF-κB expression on DPSC odontogenic differentiation in the presence of these cytokines. Thus, this study will reveal the potential for targeted disruption of inflammatory signaling in DPSCs as a potential intervention to promote pulp tissue regeneration.

## Materials and Methods

### Cell Culture Treatments

Mesenchymal human DPSCs derived from adult third molars were generously donated by Dr. Chi-Der Chen at the University of Southern California. These cells also expressed mesenchymal surface molecules such as STRO-1, CS146, SSEA4, CD73, CD105, and CD166 [26]. They were cultured in 150 cm^2^ flasks (passage 1–4) prior to seeding in 6-well plates. A cell density of 50,000 cells per square cm was used for the gene expression and collagen matrix experiments. For differentiation, media consisting of α-MEM (Invitrogen, Carlsbad, CA), 10% fetal bovine serum (VWR, Radnor, PA), and 1% pen/strep (VWR, Radnor, PA) was supplemented with 50 ppm ascorbic acid (AA; Sigma Aldrich, St. Louis, MO) and 10mM β-glycerophosphate (β-GP; Sigma Aldrich, St. Louis, MO).

The experimental treatment groups consisted of differentiation media with IL-1β +/- TNFα (R&D Systems, Minneapolis, MN) and/or MG-132 (Sigma Aldrich, St. Louis, MO) or p65 siRNA (Santa Cruz Biotech, Santa Cruz, CA). A combined dose of IL-1β at 10 ng/ml and TNFα at 20 ng/ml was used to represent a “high” dose of these inflammatory cytokines based on changes in the NF-κB translocation and expression of inflammatory-related enzymes as well as ECM degradation [27, 28]. Lower doses of these cytokines (IL-1β: 0.5 ng/ml, TNFα: 1 ng/ml) were also studied.

Transfection of p65 siRNA was accomplished with transfection media (Optimem, Life Technologies; Carlsbad, CA) and transfection reagent (Lipofectamine 2000, Life Technologies; Carlsbad, CA). The cells were transfected with a target-specific 19–25 nucleotide siRNA designed to knock down the gene expression of p65 NF-κB (sc-29410, Santa Cruz Biotechnology, Inc.). The sequence for the p65 siRNA oligonucleotide is GCCCUAUCCCUUUACGUC (Sense) and GACGUAAAGGGAUAGGGC (Anti-Sense) (Note: all sequences are provided in 5′ → 3′ orientation). The siRNAs comprising a scrambled sequence was similarly transfected as control siRNA (sc-372, Santa Cruz Biotechnology, Inc.). The exact 15–20 amino acid immunogen is proprietary but the epitope maps to the last 50 amino acids of Q04206 (residues 501–551).

Media was replaced every 2–3 days with fresh media containing the treatments. The cells were grown for 7 days in most experiments, 9 days for collagen matrix formation analysis, and 12 days for SEM analysis. Gene expression analysis was conducted using qRT-PCR after treatment at the end point of early differentiation (day 7). Concentrations of MG132 (0.1, 0.5, 1.0μM) and p65 siRNA (20 and 50 pM) were used, each 1 hour prior to cytokine exposure during separate independent studies.

MG132, IL-1β, and TNFα were added to the differentiation medium and did not require additional vehicle to affect the cells as specified in the manufacturer’s protocol. Scrambled siRNA was used to assure that the vehicle used to deliver the siRNA had a similar effect on odontogenic gene expression as the differentiation medium alone. Based on the above, the differentiation media was used as the control treatment for all experiments.

**qRT-PCR**. To quantify the levels of gene expression, qRT-PCR was prepared according to Tousi et al., 2013 [29] and Varanasi et al., 2009, 2012 [30, 31]. Using glyceraldehyde phosphate dehydrogenase (GAPDH) as an internal reference gene, the relative quantification of gene expression was evaluated using the comparative cycle threshold (CT) method and the fold change calculated using 2^−ΔΔCT^. The data were normalized to GAPDH within each independent experiment and expressed as a relative induction of control at a corresponding time-point. The National Center for Biotechnology Information (NCBI) reference sequence and accession numbers for each gene are given in Table 1.

**Table 1:** NCBI reference sequences and accession numbers of gene expression assays.

**Gene**	**NCBI Reference Sequence**	**Accession**
Human NF-κB	NM_001165412.1	NM_001165412
Human Collagen I-α1 (col(I)-α1)	NM_000088.3	NM_000088
Human Dental sialophosphoprotein (DSPP)	NM_014208.3	NM_014208
Human Nestin (Nes)	NM_006617.1	NM_006617
Human GAPDH	NM_001256799.2	NM_001256799

### Histological Analysis

Cells were seeded onto autoclaved glass cover slips and cultured for 9 days to analyze the collagen formation. Samples were fixed and prepared using Picrosirius staining as previously described [30, 31]. Images were taken using an optical microscope (Nikon Eclipse 80i) with a CCD camera and Image Pro software. Bioquant software (Bioquant Osteo; Nashville, TN) was used for relative quantitation of collagen fiber formation within the ECM in treated cells vs. control cells. The results shown represent the surface area coverage by collagen vs. the total area, following procedures outlined by Avino et al. [32] and Barron et al. [33]. Optical micrographs were imaged and captured at 100x magnification (10x eye piece, 10x objective lens).

**Chemiluminescent NF-κB p65 Transcription Factor ELISA assay**. Activation of NF-κB was assessed by the nuclear translocation of the NF-κB protein p65. p65 Transcription Factor kit (Thermo Scientific, Waltham, MA) was used to quantify active forms of p65 from the cell protein extracts according to the manufacturer’s instructions. Briefly, working binding buffer was added to appropriate streptavidin-coated wells of a 96-well plate with a bound NF-κB biotinylated-consensus sequence. Samples and controls were added for 1 hour at room temperature with mild agitation. This step was subsequently followed by the addition of primary and secondary antibodies, each for 1-hour incubation at room temperature. A chemiluminescent substrate was added, and the luminescence was read on the BIO-TEK HT luminometer/spectrophotometer.

**Intracellular Protein Extraction and Alkaline Phosphatase (ALP) assay**. The SensoLyte pNPP Alkaline Phosphatase assay kit (Anaspec, Fremont, CA) was used to extract intracellular protein and to detect intracellular alkaline phosphatase, according to the manufacturer’s instructions. Triton X-100 from the kit was used to lyse the plasma membranes of the cells, allowing intracellular proteins to be collected and measured. Intracellular alkaline phosphatase from the samples was then measured by adding pNPP substrate solution. Absorbance was measured at 405 nm on a spectrophotometer.

**Cell Viability Assay**. In a separate experiment, DPSCs were seeded (50,000 cells per sq. cm) into a 96-well plate. The cells were given the same treatments as described above. Each treatment and control group were administered to cells in triplicate, and each experiment was conducted in triplicate. At the time of the assay, the cell media was exchanged with a viability assay media using the MTS assay following the manufacturer’s instructions (Promega Inc., Madison, WI) as previously described [30]. The colorimetric assay product was then measured for the solution color change in a standard spectrophotometer to measure cell viability at 490 nm.

**SEM Imaging**. The extracellular matrices were visualized for odontoblastic morphology using scanning electron microscopy (SEM). Imaging of the cell layers was conducted using a JEOL JSM-6010LA Analytical Scanning Electron Microscope (JOEL Inc., Tokyo, Japan) operating at a voltage of 5–15kV under high vacuum.

**Statistical Analysis**. Statistical comparison of the results was made using GraphPad Prism^TM^ version 5 (Graph Pad Software Inc., San Diego, CA). Results from three independent experiments with two replications were combined for presentation as the mean ± standard error of the mean. One-way ANOVA Dunnett and t-test comparisons were used for parametric analyses. p<0.05 was considered statistically significant.

### Results

#### MG132 and p65 siRNA Reduce the NF-κB Expression in Human DPSCs Exposed to Inflammatory Cytokines

In the first part of this study, the effects of combined inflammatory cytokines (TNFα, IL-1β) on DPSC NF-κB and nuclear p65 expression were investigated. The DPSCs were induced to differentiate and exposed to the varying doses of IL-1β and TNFα. NF-κB and p65 genes were assayed using quantitative reverse transcriptase polymerase chain reaction (qRT-PCR).

**Figure 1:**
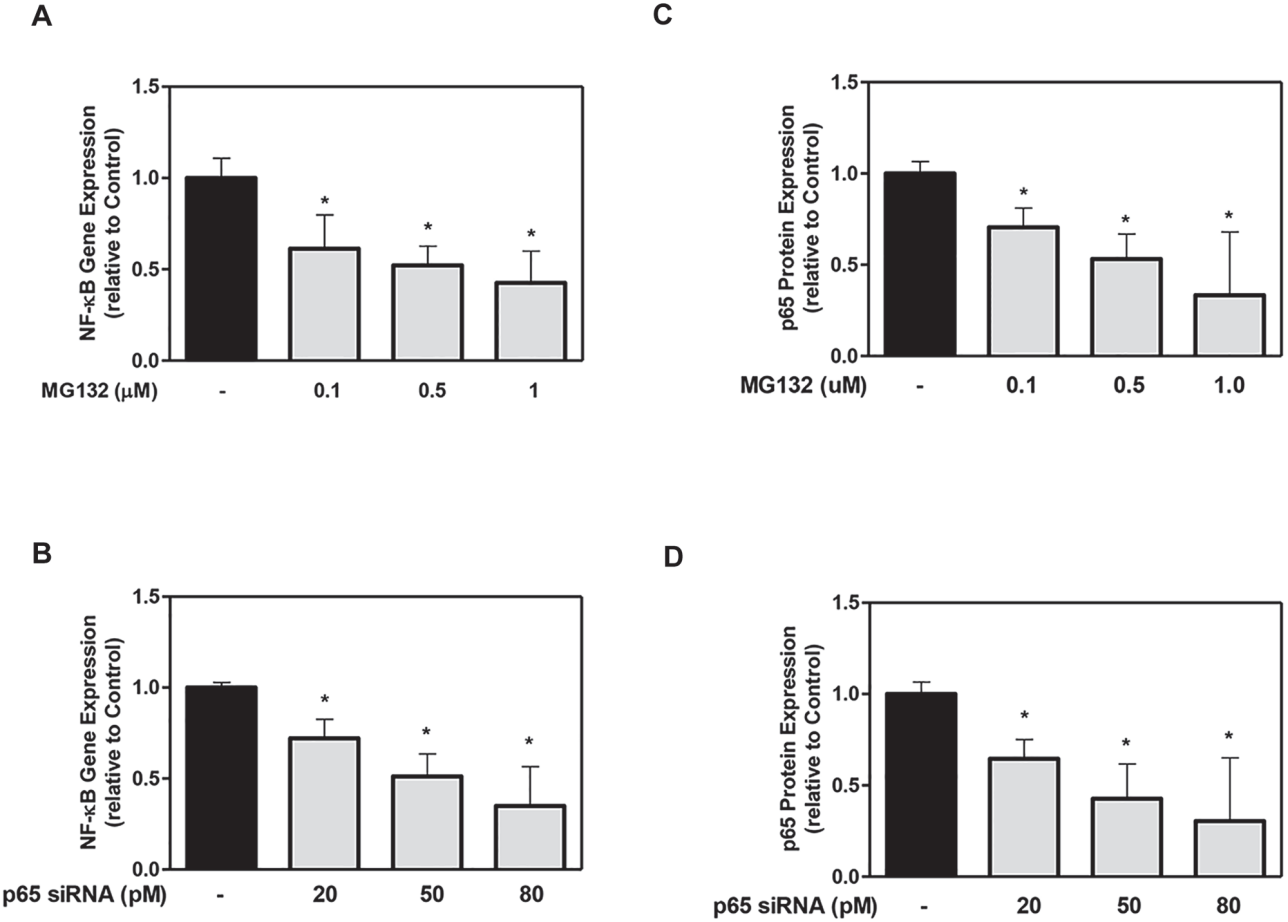
The effect of MG132 protease inhibitor and p65 siRNA on DPSC NF-κB gene expression and p65 protein expression. Cells were exposed to various MG132 or p65 siRNA doses for 7 days. NF- κB mRNA (A, B) was assayed using qRT-PCR. NF-κB p65 protein expression (C, D) was evaluated using the Chemiluminescent NF-κB p65 Transcription Factor ELISA assay:. Data are presented as the mean ± S. E. M. of triplicate measures from triplicate experiments. Symbols: Asterisks (*) indicates statistical comparison with control result. Statistical comparison was made using ANOVA testing with Dunnett’s posthoc analysis. Statistical significance was represented by * for *p* < 0.05; ** for p < 0.01; *** for p < 0.001.

The results from NF-κB gene expression analysis without cytokine addition at day 7 showed a dose-dependent decrease in the gene levels with increasing MG132 (Fig. 1A) and p65 siRNA doses (Fig. 1B) compared to the control group. The NF-κB gene expression decreased to its lowest level of 50% at a 1 μM MG-132 dose (Fig. 1A) and approximately 50% and 30% at 50 pM and 80 pM siRNA, respectively (Fig. 1B). Similarly, nuclear p65 protein levels also decreased in a dose-dependent manner by increasing the MG132 dose to its lowest level of 25% compared to the control (Fig. 1C and 1D). These results confirm that MG132 and p65 siRNA dose-dependently down-regulated the NF-κB expression.

**Figure 2:**
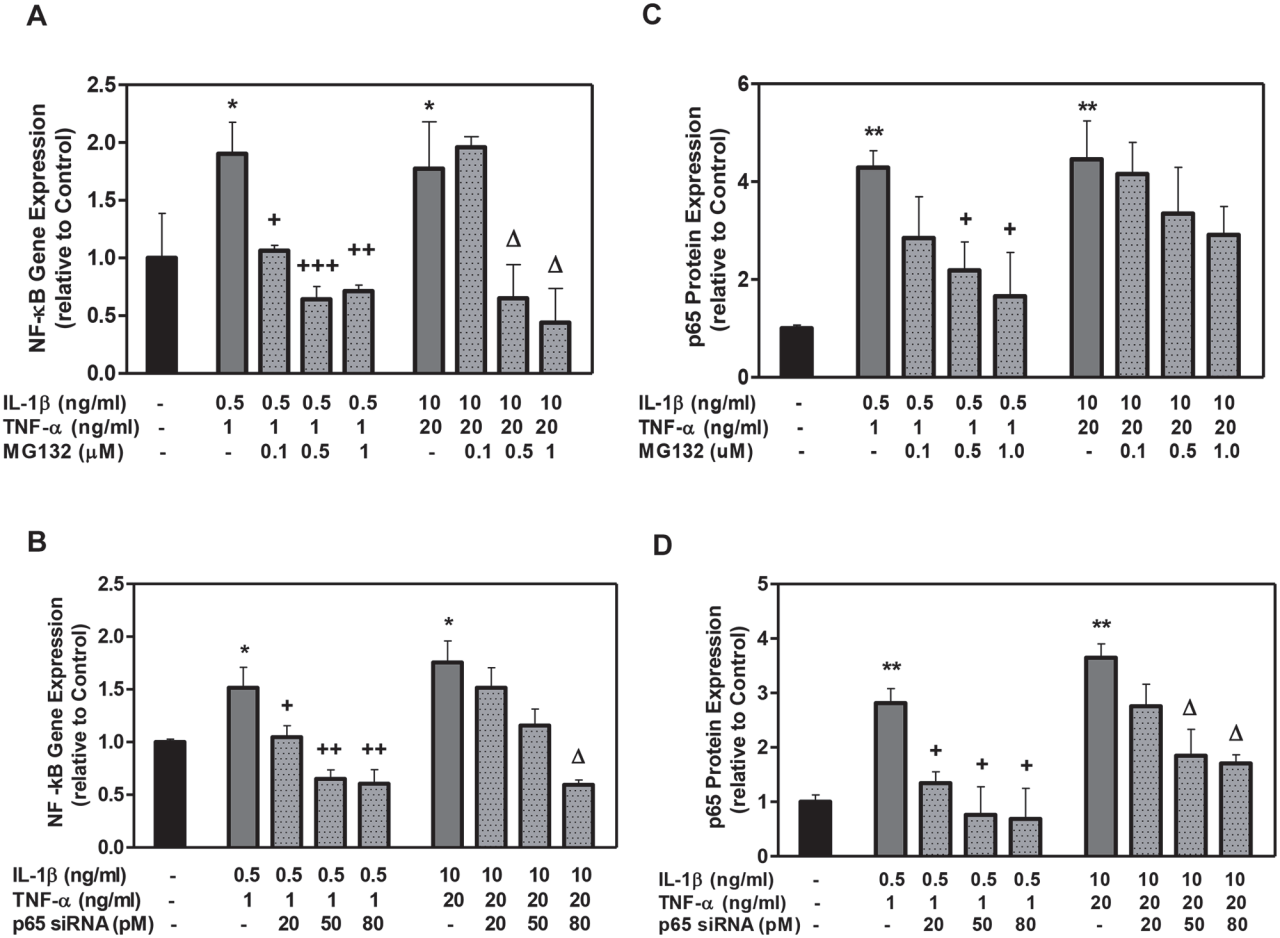
The effect of MG132 protease inhibitor and p65 siRNA on DPSC NF-κB gene expression and p65 protein expression with low- and high-cytokine doses. Cells were exposed to various MG132 or p65 siRNA concentrations for 7 days. NF-κB mRNA (A, B) was assessed using qRT-PCR. NF-κB p65 protein (C, D) was assessed using the Chemiluminescent NF-κB p65 Transcription Factor ELISA assay:. Data are presented as the mean ± S. E. M. of triplicate measures from triplicate experiments. Symbols: Asterisks (*) indicate statistical comparison with control result; plus signs (+) indicate statistical comparison with IL-1β (0.5 ng/ml) and TNFα (1.0 ng/ml) treatment (low cytokine dose); triangle (Δ) indicates statistical comparison with IL-1β (10.0 ng/ml) and TNFα (20.0 ng/ml) treatment (high cytokine dose). Statistical comparison was made using ANOVA testing with Dunnett’s posthoc analysis. Statistical significance was represented by *, +, or Δ for *p* < 0.05; **, ++, or ΔΔ for p < 0.01; ***, +++, or ΔΔΔ for p < 0.001.

IL-1β + TNFα significantly increased the NF-κB expression in the DPSCs compared to the controls (Fig. 2A, 2B, 2C, and 2D), and a dose-dependent increase in NF-κB expression was observed with increasing concentration of these cytokines. These results confirm the dose-dependent NF-κB activation by these inflammatory cytokines in DPSCs.

A dose-dependent decrease of NF-κB gene expression and nuclear p65 protein levels was induced by MG132 and p65 siRNA on day 7 (Fig. 2A, 2B, 2C, and 2D). These significant reductions ranged from a 25% to 75% drop in levels compared to each respective cytokine dose alone, demonstrating that MG132 and p65 siRNA reduce NF-κB expression in DPSCs in the presence of IL-1β and TNFα.

#### Reduction of NF-κB Levels Enhanced Odontoblastic Differentiation in the Presence of Inflammatory Cytokines

In the second part of this study, the effects of combined inflammatory cytokines (TNFα, IL-1β) on DPSC odontogenic differentiation marker expression were investigated. DSPP, Nestin, and ALP protein were assayed as markers of odontogenesis in DPSCs. These DPSCs were induced to differentiate and exposed to the varying doses of IL-1β and TNFα. DSPP, Nestin, and ALP genes were assayed using qRT-PCR. Note: the results for 50 and 80 pM treatments with or without inflammatory cytokine addition were not statistically significant; thus, the 80 pM results were not included below.

**Figure 3:**
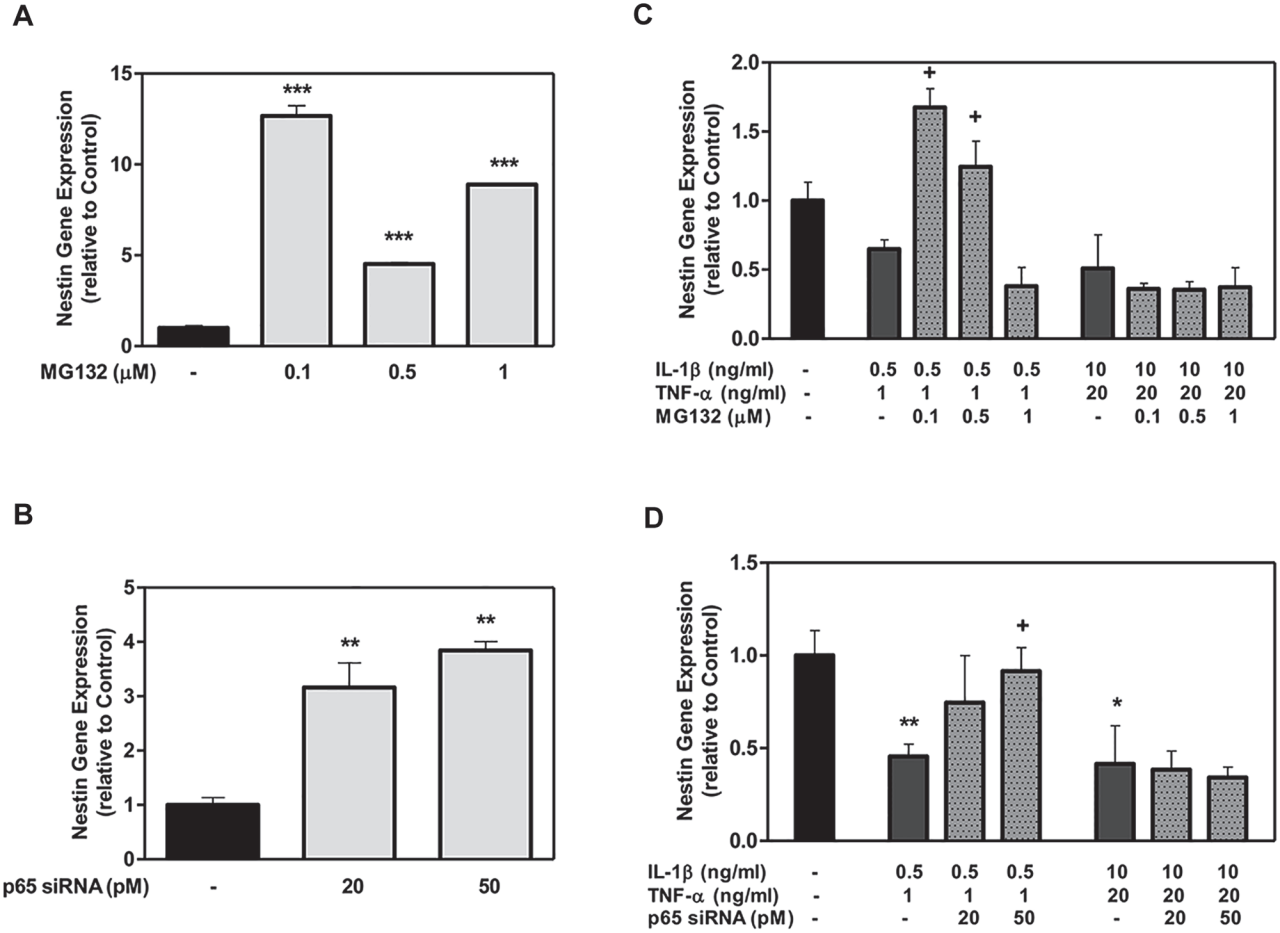
The effect of MG132 protease inhibitor and p65 siRNA on Nestin gene expression in DPSCs with low- and high-cytokine doses. DPSCs were exposed to various MG132 or p65 siRNA concentrations (A and B, respectively) and cytokines in the presence of MG132 or p65 siRNA (C and D, respectively) for 7 days. Nestin mRNA was assayed using qRT-PCR. Data are presented as the mean ± S. E. M. of triplicate measures from triplicate experiments. Symbols: Asterisks (*) indicate statistical comparison with control result; plus signs (+) indicate statistical comparison with IL-1β (0.5 ng/ml) and TNFα (1.0 ng/ml) treatment (low cytokine dose); triangle (Δ) indicates statistical comparison with IL-1β (10.0 ng/ml) and TNFα (20.0 ng/ml) treatment (high cytokine dose). Statistical comparison was made using ANOVA testing with Dunnett’s posthoc analysis. Statistical significance was represented by *, +, or Δ for *p* < 0.05; **, ++, or ΔΔ for p < 0.01; ***, +++, or ΔΔΔ for p < 0.001.

Both MG132 and p65 siRNA elevated Nestin gene expression in DPSCs in the absence of inflammatory cytokines compared to the control condition (Fig. 3A and 3B). MG132 induced a maximum enhancement of 13-fold by 0.1 μM (Fig. 3A), with siRNA also resulting in a significant enhancement of 4-fold at 50 pM (Fig. 3B). In the presence of inflammatory cytokines, the DPSCs showed a reduction in Nestin gene expression compared to the controls (Fig. 3C and 3D); however, Nestin gene expression increased when MG132 or p65 siRNA was added. This relationship was only seen at low cytokine doses, where MG132 at 0.1 and 0.5 μM and p65 siRNA at 50 pM resulted in an increase of Nestin expression by ~2-fold compared to a low cytokine dose alone (Fig. 3C and 3D). At the higher cytokine dose, MG132 and p65 siRNA did not show a similar effect.

**Figure 4:**
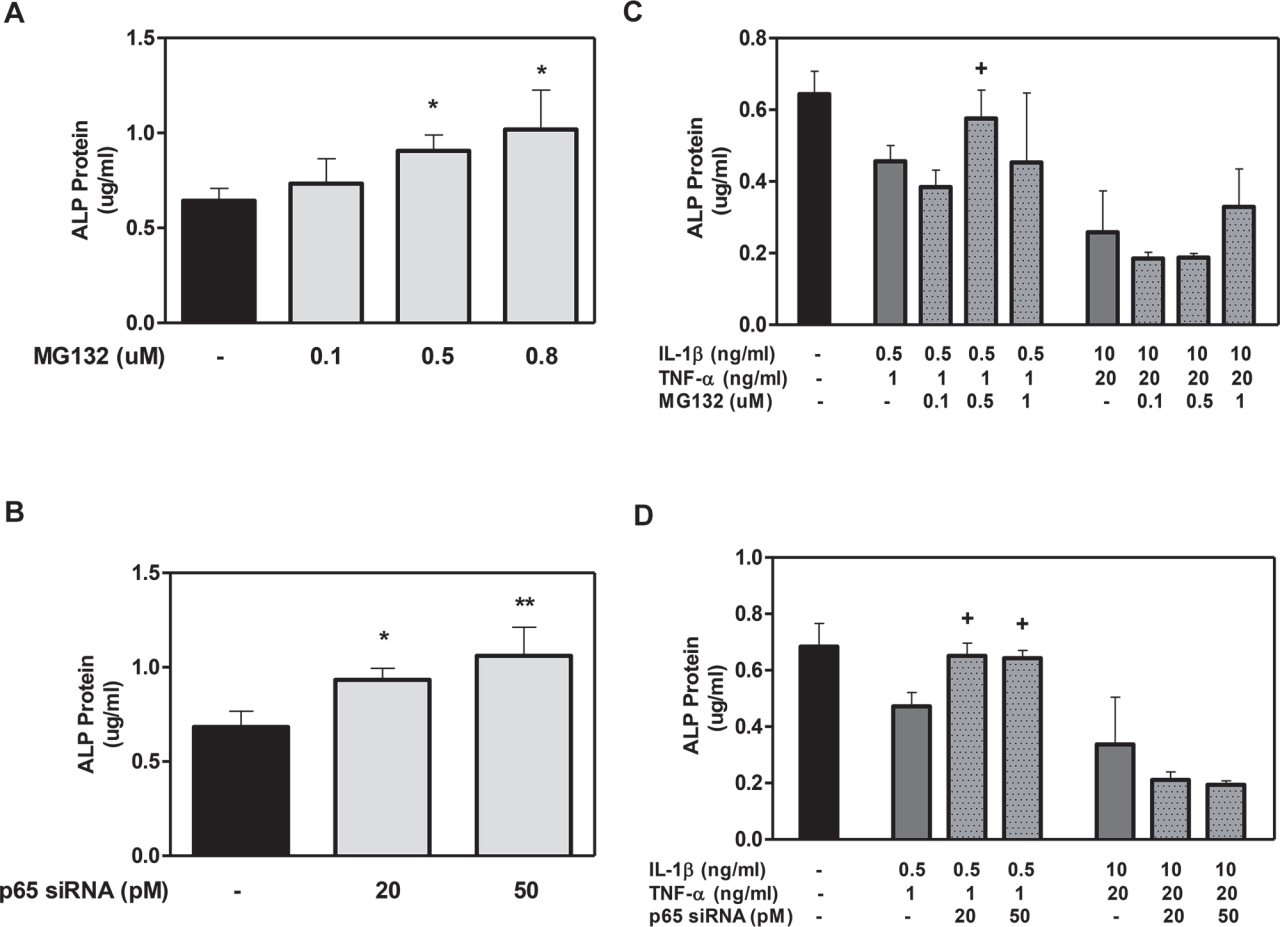
The effect of MG132 protease inhibitor and p65 siRNA on Alkaline Phosphatase (ALP) protein expression in DPSCs with low- and high-cytokine doses. DPSCs were exposed to various MG132 or p65 siRNA concentrations (A and B, respectively) and cytokines in the presence of MG132 or p65 siRNA (C and D, respectively) for 7 days. ALP protein was assayed using SensoLyte ALP Assay. Data are presented as the mean ± S. E. M. of triplicate measures from triplicate experiments. Symbols: Asterisks (*) indicate statistical comparison with control result; plus signs (+) indicate statistical comparison with IL-1β (0.5 ng/ml) and TNFα (1.0 ng/ml) treatment (low cytokine dose); triangle (Δ) indicates statistical comparison with IL-1β (10.0 ng/ml) and TNFα (20.0 ng/ml) treatment (high cytokine dose). Statistical comparison was made using ANOVA testing with Dunnett’s posthoc analysis. Statistical significance was represented by *, +, or Δ for *p* < 0.05; **, ++, or ΔΔ for p < 0.01; ***, +++, or ΔΔΔ for p < 0.001.

MG132 and p65 siRNA showed a similar trend in ALP protein production by DPSCs (Fig. 4). The ALP levels were significantly increased in the presence of the MG132/p65 siRNA compared to the control (Fig. 4A and 4B). Although inflammatory conditions led to a decrease in ALP protein levels compared to the control, MG132 at 0.5 μM and p65 siRNA at 20 and 50 pM increased these levels at low cytokine doses (Fig. 4C and 4D). At the higher cytokine doses, MG132/p65 siRNA produced no significant changes.

**Figure 5:**
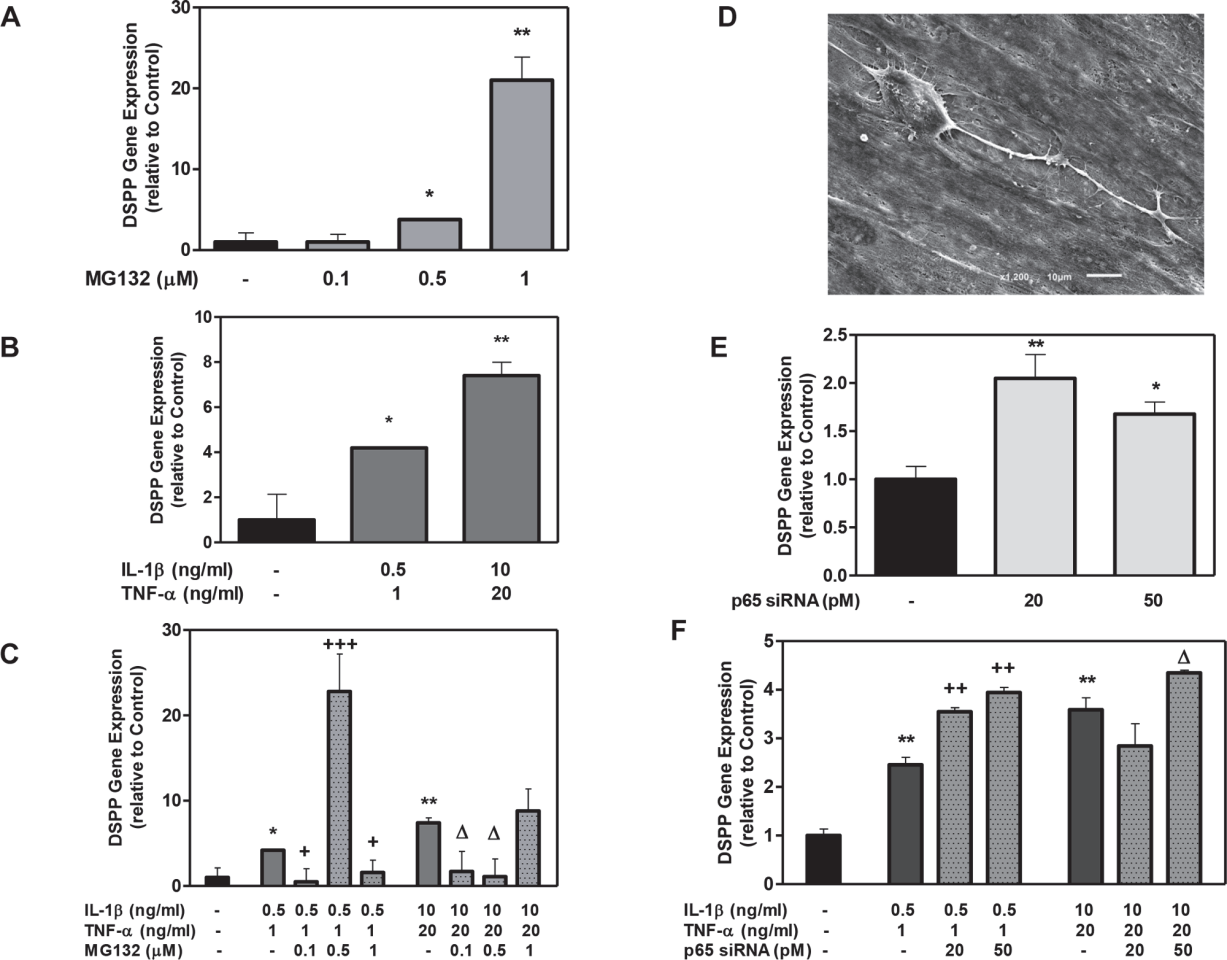
The effect of MG132 protease inhibitor and p65 siRNA on Dentin Sialophosphoprotein (DSPP) gene expression in DPSCs with low- and high-cytokine doses. DPSCs were exposed to various MG132 or p65 siRNA (A and E, respectively), cytokine dose alone (B), and cytokine dose in the presence of MG132 or p65 siRNA (C and F, respectively) for 7 days for the examination of odontoblastic gene marker DSPP. The odontoblastic cell morphology was examined using scanning electron microscopy after 12 days (D) (for treatment IL-1β (0.5) + TNFα (1) + MG132 (0.5)). DSPP mRNA was assessed using RT-PCR. Data are presented as the mean + S. E. M. of triplicate measures from triplicate experiments. Symbols: Asterisks (*) indicate statistical comparison with control result; plus signs (+) indicate statistical comparison with IL-1β (0.5 ng/ml) and TNFα (1.0 ng/ml) treatment (low cytokine dose); triangle (Δ) indicates statistical comparison with IL-1β (10.0 ng/ml) and TNFα (20.0 ng/ml) treatment (high cytokine dose). Statistical comparison was made using ANOVA testing with Dunnett’s posthoc analysis. Statistical significance was represented by *, +, or Δ for p < 0.05; **, ++, or ΔΔ for p < 0.01; ***, +++, or ΔΔΔ for p < 0.001.

The DSPP gene level was dose-dependently up-regulated at day 7 by MG132 (4–22 fold; Fig. 5A) or by both cytokine doses (4–8 fold; Fig. 5B). When 0.5 μM MG132 was added to a low cytokine dose (IL-1β at 0.5 ng/ml + TNFα at 1ng/ml), the gene expression of DSPP was enhanced by more than 6 times compared to the combined cytokine in the absence of the MG132 (Fig. 5C). This increase in DSPP expression was only observed under a low cytokine dose. Under the same treatment, SEM imaging displayed phenotypic characteristics of odontoblastic cells and a columnar cell body with 1–2 processes (Fig. 5D).

The addition of p65 siRNA also increased the DSPP gene expression by 1.5 to 2 fold without any cytokine addition (Fig. 5E). A similar increase in DSPP expression occurred when p65 siRNA was added (20 or 50 pM) to the DPSCs treated with low and high cytokine doses compared to the DPSCs treated with each cytokine dose alone (Fig. 5F). Taken together, these results illustrate that the odontogenic phenotype is enhanced in DPSCs in the presence of a low cytokine dose and NF-κB knockdown.

#### NF-κB Knockdown Enhanced DPSC Collagen Gene Expression and Matrix Synthesis

Continuing on from the second part of this study, the effects of combined inflammatory cytokines (TNFα, IL-1β) on DPSC collagen (Col(I)α1) expression and collagen matrix formation were investigated. Collagen gene expression was assayed using qRT-PCR as a marker of odontogenesis in DPSCs. The Extracellular Matrix (ECM) collagen formation was imaged and analyzed for matrix density using Picrosirius staining under polarized light microscopy.

**Figure 6:**
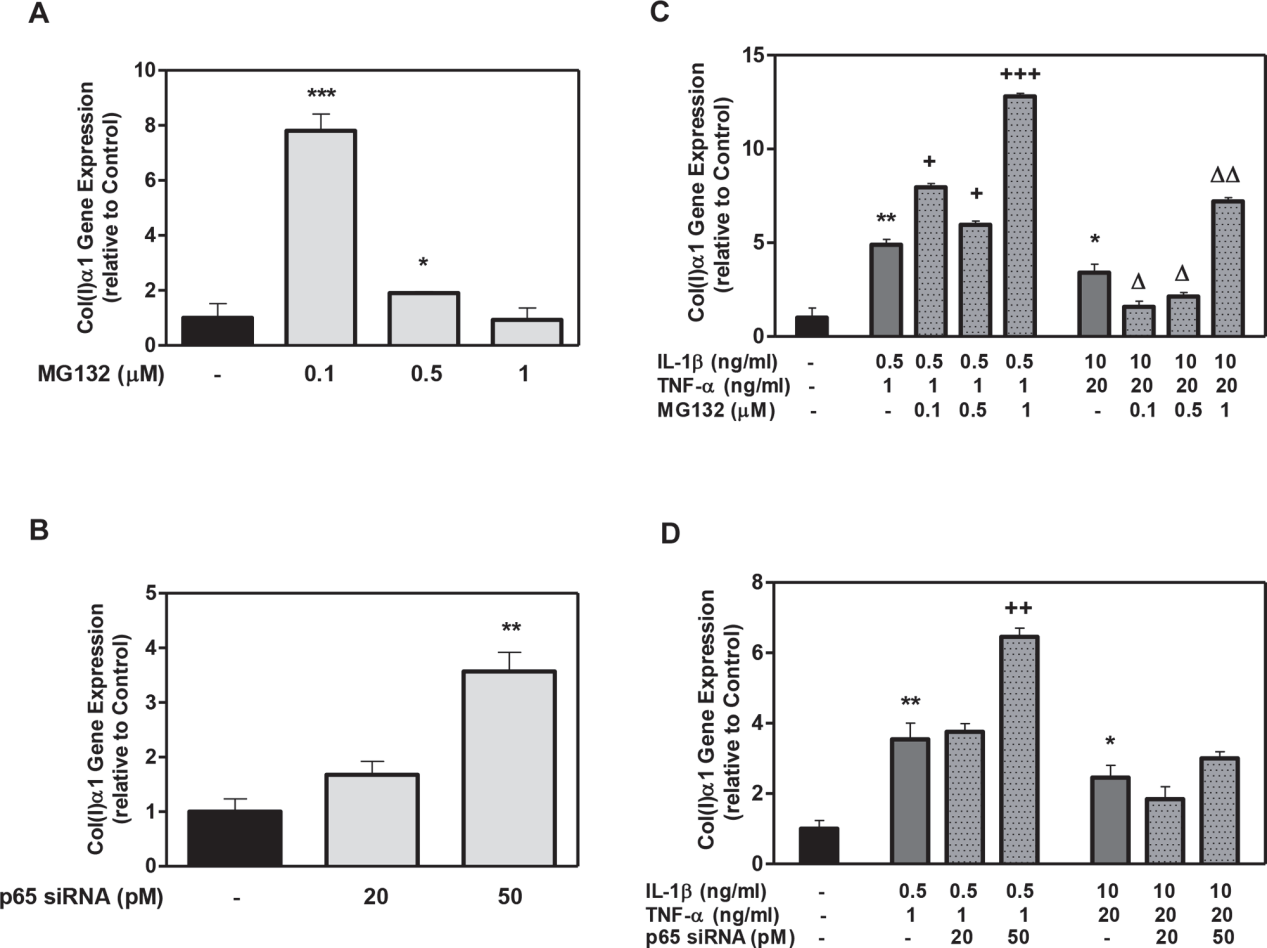
The effect of MG132 protease inhibitor and p65 siRNA on Collagen (I)-α1 (Col(I)α1) gene expression in DPSCs with low- and high-cytokine doses. DPSCs were exposed to various MG132 or p65 siRNA concentrations (A and B, respectively) and cytokines in the presence of MG132 or p65 siRNA (C and D, respectively) for 7 days. Col(I)-α1 mRNA was assayed using qRT-PCR. Data are presented as the mean ± S. E. M. of triplicate measures from triplicate experiments. Symbols: Asterisks (*) indicate statistical comparison with control result; plus signs (+) indicate statistical comparison with IL-1β (0.5 ng/ml) and TNFα (1.0 ng/ml) treatment (low cytokine dose); triangle (Δ) indicates statistical comparison with IL-1β (10.0 ng/ml) and TNFα (20.0 ng/ml) treatment (high cytokine dose). Statistical comparison was made using ANOVA testing with Dunnett’s posthoc analysis. Statistical significance was represented by *, +, or Δ for *p* < 0.05; **, ++, or ΔΔ for p < 0.01; ***, +++, or ΔΔΔ for p < 0.001.

The results for type I collagen gene expression showed that the addition of MG132 to the DPSCs induced a ~8-fold and 2-fold increase in collagen expression with 0.1μM and 0.5μM MG132, respectively, compared to the control (Fig. 6A). P65 siRNA also elevated the collagen gene expression, producing a 1.5-fold and 3.5-fold increase in collagen (I) gene expression with 20 pM and 50 pM, respectively (Fig. 6B).

Both cytokine doses significantly increased the collagen gene expression compared to the control (Fig. 6C and 6D), although this enhancement diminished with increasing cytokine dose. The subsequent addition of MG132 at low and high cytokine doses further enhanced the collagen expression relative to each respective cytokine dose alone (Fig. 6C). Similar enhanced collagen expression was observed for DPSCs exposed to p65 siRNA in the presence of the varied cytokine dose (Fig. 6D).

**Figure 7:**
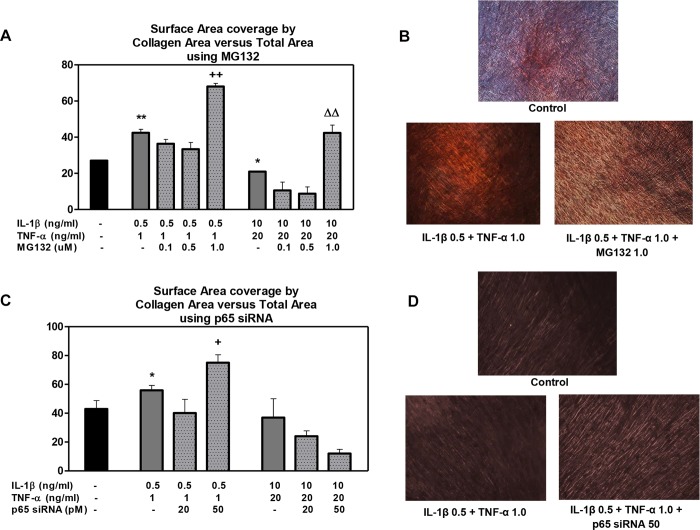
The effect of MG132 protease inhibitor and p65 siRNA on collagen matrix formation in DPSCs with low- and high-cytokine doses. Bioquant analysis of collagen formation DPSC after 9 days’ treatment. DPSCs exposed to various inflammatory treatments in the presence or absence of MG132 (A and B) or p65 siRNA (C and D) were analyzed from picrosirius staining images using the Bioquant software. Data are presented as the mean ± S. E. M. of triplicate measures from triplicate experiments. Symbols: Asterisks (*) indicate statistical comparison with control result; plus signs (+) indicate statistical comparison with IL-1β (0.5 ng/ml) and TNFα (1.0 ng/ml) treatment (low cytokine dose); triangle (Δ) indicates statistical comparison with IL-1β (10.0 ng/ml) and TNFα (20.0 ng/ml) treatment (high cytokine dose). Statistical comparison was made using ANOVA testing with Dunnett’s posthoc analysis. Statistical significance was represented by *, +, or Δ for *p* < 0.05; **, ++, or ΔΔ for p < 0.01; ***, +++, or ΔΔΔ for p < 0.001.

The collagen matrix formation was assessed on day 9 using Bioquant software analysis of picrosirius staining (Fig. 7, S1 Fig., and S2 Fig.). The DPSCs treated with a low cytokine dose showed increased collagen density compared with the DPSC-treated controls. Analogous with collagen gene expression, the collagen matrix density decreased with increasing cytokine dose. The addition of MG132 (1.0 μM) to DPSCs treated with high or low cytokine doses further enhanced the collagen density (~2-fold) relative to the DPSCs treated with each respective cytokine dose alone (Fig. 7B).

The p65 siRNA (50 pM) did enhance collagen matrix density when the DPSCs were exposed to low cytokine doses (Fig. 7C) compared to the low cytokine dose alone. These data demonstrate that MG132 and siRNA have an enhancing effect on collagen expression and formation by DPSCs in the presence of IL-1β and TNF-a.

### Discussion

The exposure of DPSCs to the inflammatory cytokines TNFα and IL-1β impairs their ability to differentiate into odontoblasts, consistent with the role of these cytokines in inhibiting dentin and pulp repair during inflammation. Because both TNFα and IL-1β increase the NF-κB-mediated gene expression in DPSCs, we tested the hypothesis that the inhibition of NF-κB activity increases odontogenic capability in these cells.

The TNFα/IL-1β treatment of DPSC cultures in this study increased the NF-κB gene expression and decreased the expression of the odontoblast markers Nestin and ALP. The inhibition of NF-κB activity significantly decreased NF-κB gene expression and increased Nestin mRNA, ALP, DSPP, and collagen expression, even though we only achieved a 60–75% reduction in nuclear p65 with either MG132 treatment or p65 knockdown. We predict that complete ablation of p65 (e.g., by gene knockout) would yield even more dramatic results.

The cytokine doses (“low” and “high”) used in this study were chosen based on previous in vitro and in vivo studies wherein similar concentrations of TNFα and IL-1β increased the NF-κB activity. Cultures of human DPSC and gingival fibroblasts/epithelial cells showed 2–4 fold activation of NF-κB with a single dose of either TNFα (10 ng/ml) or IL-1β (10 ng/ml), respectively [34, 35]. In vivo studies of mice treated with TNFα and IL-1β (4μg/kg) showed increased NF-κB activity [36], and clinical studies of primary fibroblast-like synoviocytes isolated from the synovium of patients with rheumatoid arthritis and osteoarthritis also displayed activated NF-κB signaling pathways (by ~2-fold) in the presence of TNFα (1–100ng/ml) and IL-1β (0.1–10 ng/ml) [37, 38]. We observed a similar 2–4 fold increase in NF-κB expression in our cultures (Fig. 2) and thus concluded that the DPSC culture system used in this study is a valid model of NF-κB inflammatory signaling. Indeed, we observed that the elevation of NF-κB in our low cytokine dose was nearly as much as our high dose. This finding suggests that NF-κB signaling in our DPSC cultures was especially sensitive to inflammatory cytokines.

We were surprised to see that our cytokine treatment of DPSC cultures increased DSPP and collagen expression on its own (Figs. 5, 6, and 7). Previous studies reported that short-term exposure of DPSCs to TNFα/IL-1β caused a transient increase in DSPP that gave way to a decrease with longer exposure [8–13]. It is possible that our culture conditions recreated and maintained some aspects of this “shorter-term” cytokine effect even as long as the week-long culture period of our experiments. Nevertheless, our data showed a notable increase in markers of the odontoblastic phenotype when we reduced NF-κB in the DPSCs. Thus, our results support the utility of NF-κB inhibition as a pharmacological “pressure point” to reduce the inhibitory effects of inflammation on odontogenesis.

We observed that the effects of our treatments on odontoblast marker expression varied, depending on the marker. Nestin, ALP, and collagen gene expression were all suppressed by higher doses of cytokines versus lower ones (Figs. 3, 4, 6, and 7). Simplistically, we attributed this phenomenon to a reduction in cell viability in the presence of high levels of inflammatory signals (S3 Fig.). However, DSPP expression was increased by a higher cytokine dose over that seen in the lower dose (Fig. 5), suggesting that the inflammatory signal (Nf-κB-mediated or other) in the surviving cells overcame any reduction caused by the presence of fewer viable cells in the culture. Similarly, we found that higher levels of MG132 in the culture decreased cell viability (S3 Fig.), which is perhaps not surprising for a generalized proteosome inhibitor. Nevertheless, ALP and collagen expression were elevated in some cases by the highest concentration of MG132 over lower concentrations (Figs. 4C and 6C), suggesting that the effect of this inhibitor on odontoblast marker expression was even more potent in the remaining viable cells than our data revealed. We observed a similar varied pattern from gene to gene with p65 knockdown. Nestin, ALP, and collagen levels were all suppressed by the high cytokine dose (Figs. 3, 4, and 6), but DSPP was not (Fig. 5). Rather, DSPP was mildly elevated by p65 knockdown at a high cytokine dose, and then only by one concentration of p65 siRNA (Fig. 5F). This result indicates that other NF-κB-independent signals transduced in the cells in response to cytokine treatment superseded the NF-κB-dependent ones. Thus, the variations in response to what we observed from gene to gene suggest differences in the importance of NF-κB or inflammatory signals for individual odontoblast gene expression.

The connections between NF-κB and dentin function remain unclear. NF-κB may directly activate the expression of odontoblast genes to control odontoblast differentiation. It may also indirectly control the generation of mature dentin ECM. TNFα and IL-1β increase the production of MMPs in chondrocytes via NF-κB signaling, which has been implicated in collagen degradation in osteoarthritis [39] and dental pulp [14]. Other studies have shown a decrease in the production of MMPs via the inhibition of NF-κB by MG132 the [40], possibly allowing for enhanced collagen deposition.

Ongoing studies will focus on the mechanism of NF-κB action in odontoblast and dentin generation.

### Conclusions

In this study, we tested the hypothesis that decreased NF-κB expression in the presence of elevated inflammatory cytokine levels can enhance DPSC odontogenic differentiation and collagen matrix formation. Elevated cytokine levels increased NF-κB expression while moderately enhancing DSPP and collagen expression. Yet, the Nestin and ALP expression and collagen matrix density decreased with increasing cytokine dose. The knockdown of NF-κB at these elevated cytokine levels enhanced all DPSC odontogenic markers, including a marked enhancement of collagen matrix formation, DSPP expression, and expression of the odontoblastic phenotype. Thus, this study showed that using NF-κB knockdown as a possible intervention for inflammatory signaling can enhance DPSC odontogenesis to potentially enhance pulp tissue regeneration.

## Supporting Information

S1 Fig DPSC collagen matrix formation with low- and high-cytokine doses and knockdown (MG132) of NF-κB. Picrosirius staining images of DPSC after 9 days’ treatment. Picrosirius staining was conducted to look at ECM (collagen) matrix formation. A: Control, B: IL-1β (0.5 ng/ml) + TNFα (1ng/ml), C: IL-1β (0.5) + TNFα (1) + MG132 (0.1μM), D: IL-1β (0.5) + TNFα (1) + MG132 (0.5), E: IL-1β (0.5) + TNFα (1) + MG132 (1), F: IL-1β (10) + TNFα (20), G: IL-1β (10) + TNFα (20) + MG132 (0.1), H: IL-1β (10) + TNFα (20) + MG132 (0.5), I: IL-1β (10) + TNFα (20) + MG132 (1). Note: All images were captured at 100x magnification.
(TIF)

S2 Fig DPSC collagen matrix formation with low- and high-cytokine doses and knockdown (p65 siRNA) of NF-κB. Picrosirius staining images of DPSC after 9 days’ treatment. Picrosirius staining was conducted to look at ECM (collagen) matrix formation. A: Control, B: IL-1β (0.5 ng/ml) + TNFα (1ng/ml), C: IL-1β (0.5) + TNFα (1) + p65 siRNA (20pM), D: IL-1β (0.5) + TNFα (1) + p65 siRNA (50 pM), F: IL-1β (10) + TNFα (20), G: IL-1β (10) + TNFα (20) + p65 siRNA (20pM), H: IL-1β (10) + TNFα (20) + p65 siRNA (50 pM). Note: All images were captured at 100x magnification.
(TIF)

S3 Fig DPSC viability with low- and high-cytokine doses and MG132 protease inhibitor and p65 siRNA. Cells were exposed to IL-1β (0.5–10 ng/ml) and TNFα (1–20 ng/ml) in the presence or absence of MG132 (0.1–1 μM) (A) or p65 siRNA (20–80 pM) (B) for 7 days. Cell viability was assayed using the MTS assay. Data are presented as the mean ± S. E. M. of triplicate measures from triplicate experiments. Symbols: Asterisks (*) indicate statistical comparison with control result; plus signs (+) indicate statistical comparison with IL-1β (0.5 ng/ml) and TNFα (1.0 ng/ml) treatment (low cytokine dose); triangle (Δ) indicates statistical comparison with IL-1β (10.0 ng/ml) and TNFα (20.0 ng/ml) treatment (high cytokine dose). Statistical comparison was made using ANOVA testing with Dunnett’s posthoc analysis. Statistical significance was represented by *, +, or Δ for *p* < 0.05; **, ++, or ΔΔ for p < 0.01; ***, +++, or ΔΔΔ for p < 0.001.
(TIF)
