# Correction: Decreasing NF-κB Expression Enhances Odontoblastic Differentiation and Collagen Expression in Dental Pulp Stem Cells Exposed to Inflammatory Cytokines

**DOI:** 10.1371/journal.pone.0127494

**Published:** 2015-04-29

**Authors:** Neda S. T. Hozhabri, M. Douglas Benson, Michael D. Vu, Rinkesh H. Patel, Rebecca M. Martinez, Fatemeh N. Nakhaie, Harry K. W. Kim, Venu G. Varanasi

The following information is missing from the Funding section: The authors would also like to thank the National Institutes of Health / National Institutes for Dental and Craniofacial Research for their support (Grant 1R03DE023872-02, Varanasi, PI).
