# Supplementary figures and images for: Decreasing NF-κB Expression Enhances Odontoblastic Differentiation and Collagen Expression in Dental Pulp Stem Cells Exposed to Inflammatory Cytokines
Source: PLoS One. 2015 Jan 28;10(1):e0113334. doi: 10.1371/journal.pone.0113334 (PMC4309458; doi:10.1371/journal.pone.0113334)

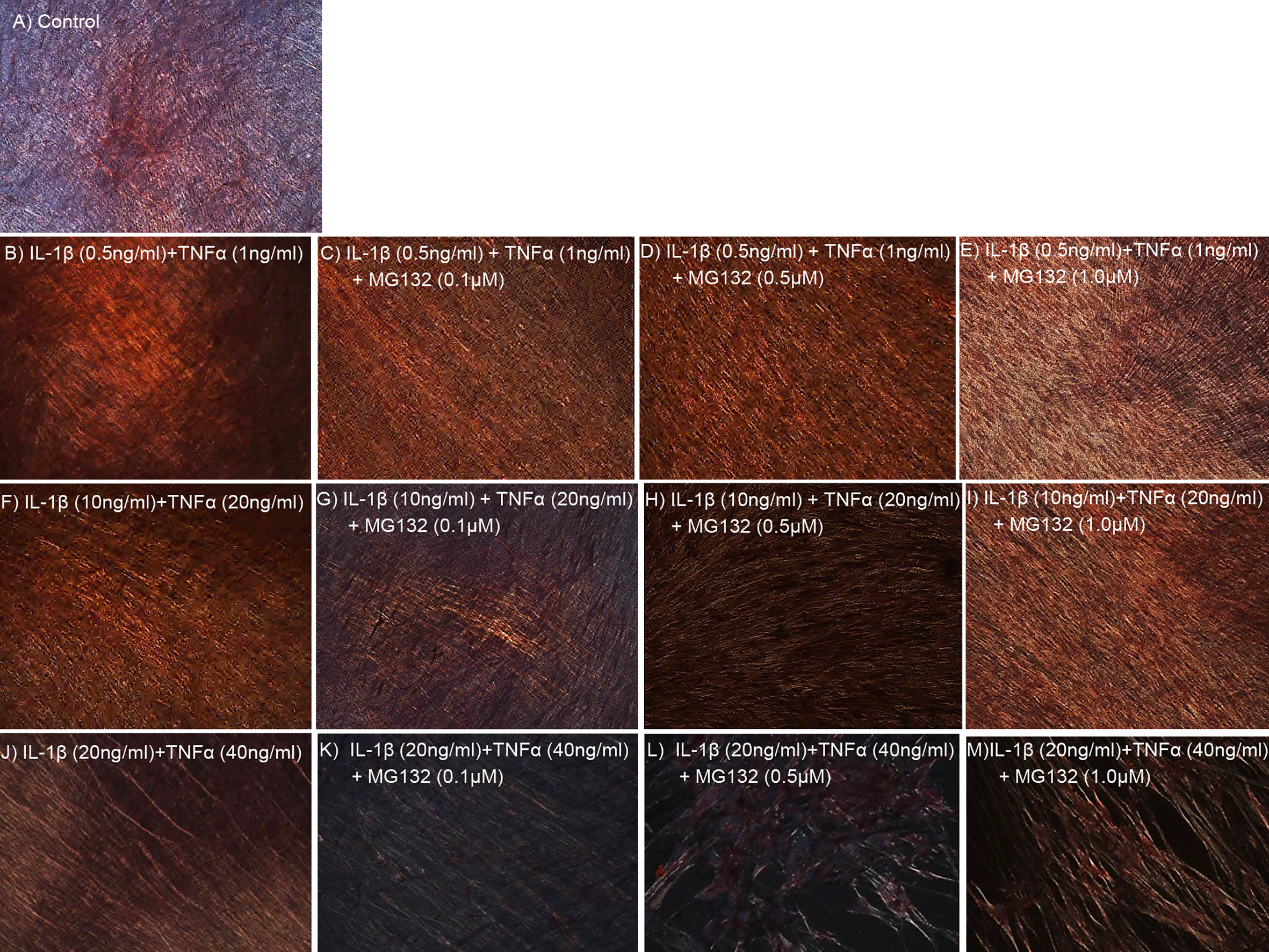

Supplement: S1 Fig — Picrosirius staining images of DPSC after 9 days’ treatment. Picrosirius staining was conducted to look at ECM (collagen) matrix formation. A: Control, B: IL-1β (0.5 ng/ml) + TNFα (1ng/ml), C: IL-1β (0.5) + TNFα (1) + MG132 (0.1μM), D: IL-1β (0.5) + TNFα (1) + MG132 (0.5), E: IL-1β (0.5) + TNFα (1) + MG132 (1), F: IL-1β (10) + TNFα (20), G: IL-1β (10) + TNFα (20) + MG132 (0.1), H: IL-1β (10) + TNFα (20) + MG132 (0.5), I: IL-1β (10) + TNFα (20) + MG132 (1). Note: All images were captured at 100x magnification. (TIF) [file pone.0113334.s001.tif]

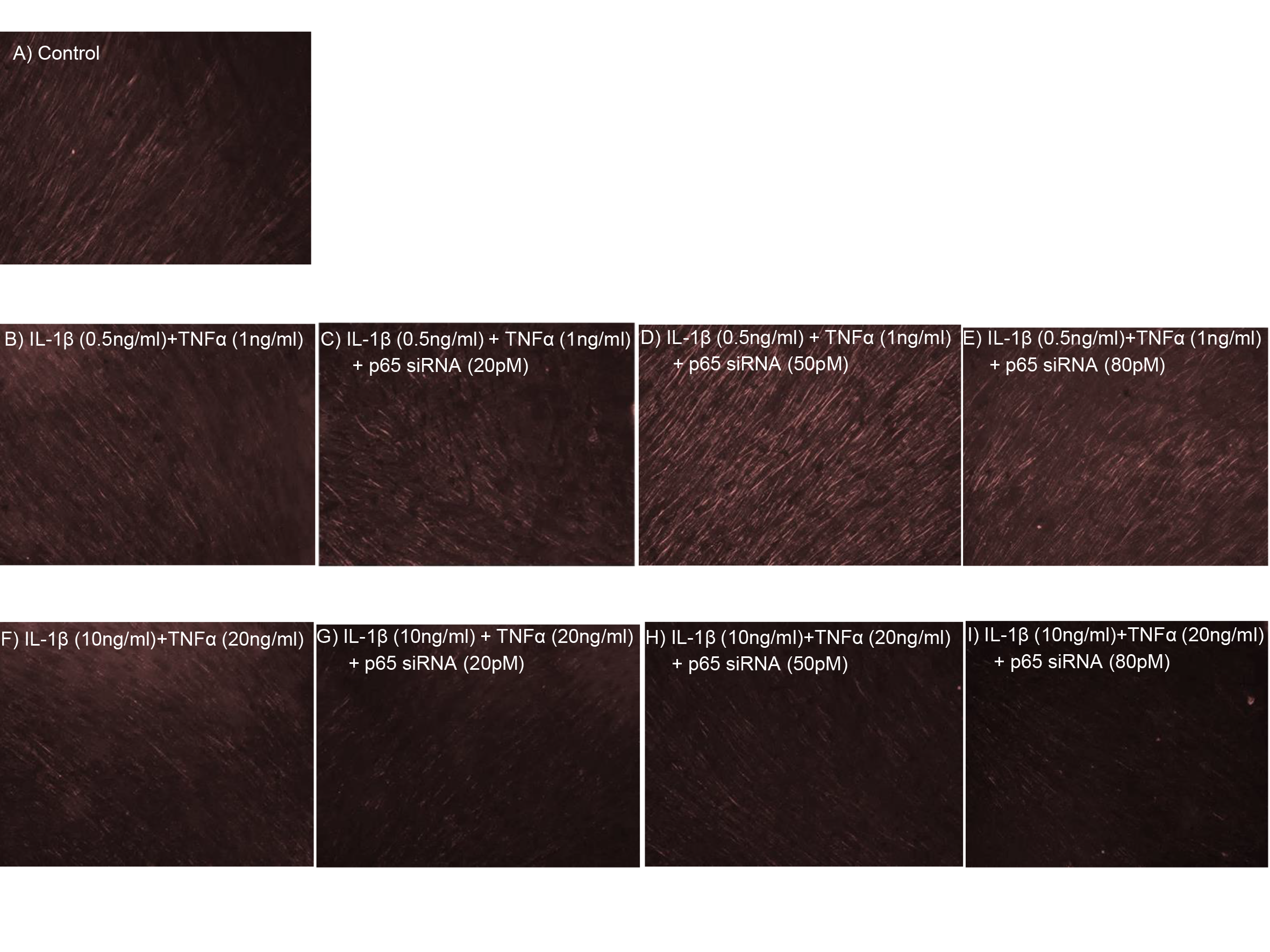

Supplement: S2 Fig — Picrosirius staining images of DPSC after 9 days’ treatment. Picrosirius staining was conducted to look at ECM (collagen) matrix formation. A: Control, B: IL-1β (0.5 ng/ml) + TNFα (1ng/ml), C: IL-1β (0.5) + TNFα (1) + p65 siRNA (20pM), D: IL-1β (0.5) + TNFα (1) + p65 siRNA (50 pM), F: IL-1β (10) + TNFα (20), G: IL-1β (10) + TNFα (20) + p65 siRNA (20pM), H: IL-1β (10) + TNFα (20) + p65 siRNA (50 pM). Note: All images were captured at 100x magnification. (TIF) [file pone.0113334.s002.tif]

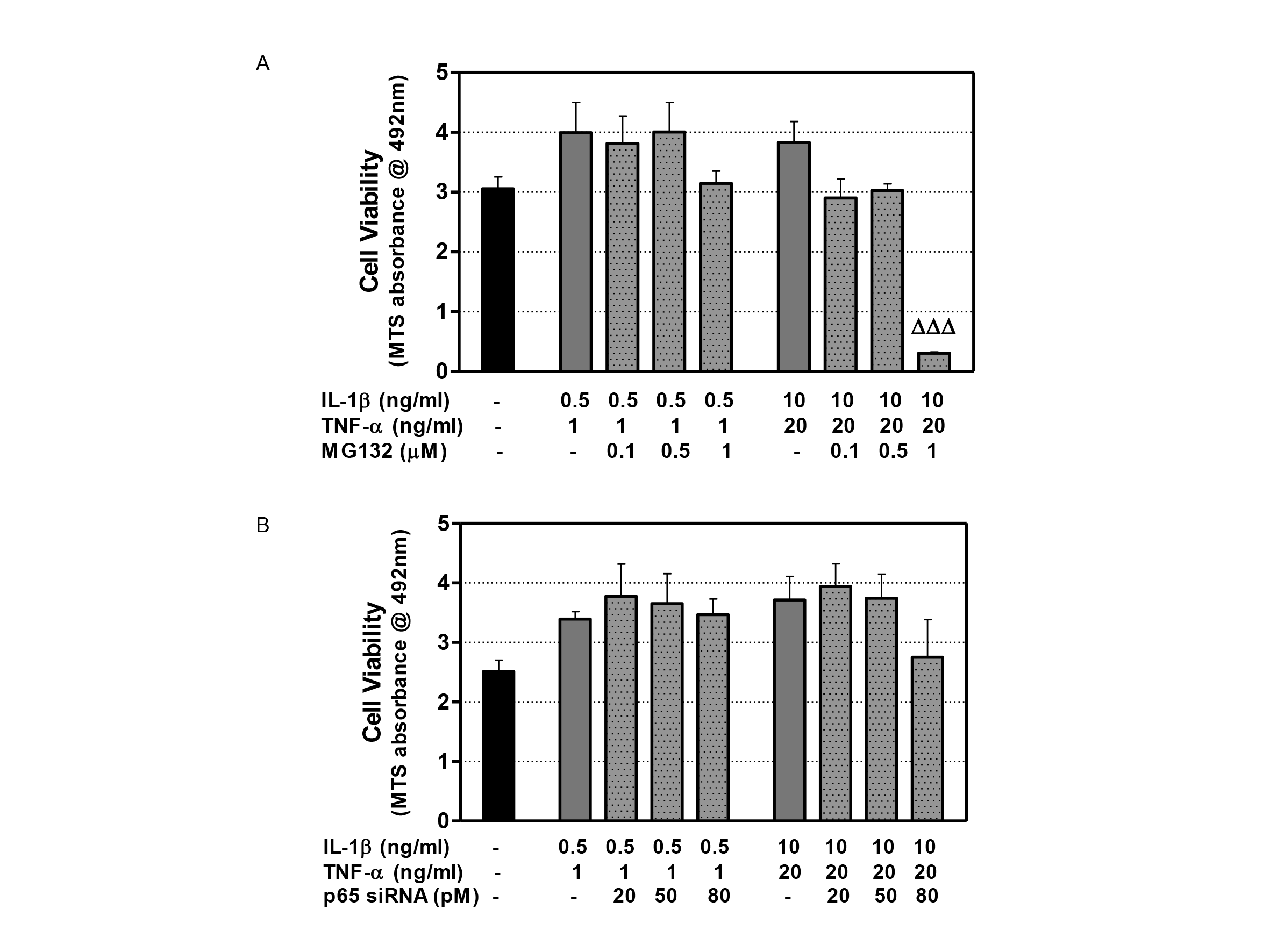

Supplement: S3 Fig — Cells were exposed to IL-1β (0.5–10 ng/ml) and TNFα (1–20 ng/ml) in the presence or absence of MG132 (0.1–1 μM) (A) or p65 siRNA (20–80 pM) (B) for 7 days. Cell viability was assayed using the MTS assay. Data are presented as the mean ± S. E. M. of triplicate measures from triplicate experiments. Symbols: Asterisks (*) indicate statistical comparison with control result; plus signs (+) indicate statistical comparison with IL-1β (0.5 ng/ml) and TNFα (1.0 ng/ml) treatment (low cytokine dose); triangle (Δ) indicates statistical comparison with IL-1β (10.0 ng/ml) and TNFα (20.0 ng/ml) treatment (high cytokine dose). Statistical comparison was made using ANOVA testing with Dunnett’s posthoc analysis. Statistical significance was represented by *, +, or Δ for p < 0.05; **, ++, or ΔΔ for p < 0.01; ***, +++, or ΔΔΔ for p < 0.001. (TIF) [file pone.0113334.s003.tif]
